# Acute Non-glue Pulmonary Embolism Following Endoscopic Ultrasound (EUS)-Guided Glue/Coil Treatment for Gastric Varices: A Case Report and Literature Review

**DOI:** 10.7759/cureus.27446

**Published:** 2022-07-29

**Authors:** Ahmed H Abdelfattah, Usama Talib, Ahmed N Elkot, Hadeel Dawoud, Amaar Talib

**Affiliations:** 1 Internal Medicine/Hospital Medicine, University of Kentucky College of Medicine, Lexington, USA; 2 Internal Medicine, University of Kentucky College of Medicine, Lexington, USA; 3 Gastroenterology and Hepatology, Mansoura University Hospital, Mansoura, EGY; 4 Critical Care Medicine, Mansoura University, Mansoura, EGY; 5 Orthopaedics, Hijaz Hospital, Lahore, PAK

**Keywords:** non glue pe, cardiovascular, endoscopic ultrasound (eus), esophagogastroduodenoscopy (egd), glue injection related pe, glue pe, glue injection, pulmonary embolism (pe), pe, esophageal and gastric varices

## Abstract

Decompensated liver cirrhosis (DLC) is sometimes associated with the development of esophageal varices (EV) and gastric varices (GV). GV is less common than EV. One of the treatment methods for GV is the injection of glue into the varices, which can be complicated by the embolism of the glue into the pulmonary vessels called glue pulmonary embolism (GPE). Non-glue pulmonary embolism (NGPE) after treatment of gastric varices is not very commonly reported in the literature. Herein, we present a case of the development of non-GPE after the treatment of the GV with glue injection and coiling.

## Introduction

Over the last decade, our understanding of the nature of liver diseases has evolved. The previous understanding of a state of natural “auto-anticoagulation” (increased bleeding risk) has evolved into a new term called “rebalanced hemostasis” [1-2]. This is due to simultaneous changes and decreased synthesis of pro and anticoagulation factors [2]. Decompensated liver cirrhosis (DLC) can be complicated by the development of esophageal varices (EV) and gastric varices (GV). The incidence of GV in DLC has been estimated to be around 20% [3]. Gastric varices can be treated by different modalities, either endoscopically or radiologically. One of the most common methods is the use of a cyanoacrylate injection also known as glue therapy (GT). Glue therapy has been reported in some cases to be complicated by the development of glue-related pulmonary embolisms (GPE). Many risk factors for the development of GPE have been identified in the literature, such as the volume of the injected glue, the speed of injection, and the size of the gastric varices [4]. In our case, we present the development of regular (blood clot-related) pulmonary embolism (PE), also known as non-glue PE (NGPE). This occurred after endoscopic treatment of the gastric varices with glue injection. Contrary to the treatment of GPE, our patient was treated with anticoagulation despite a recent episode of gastrointestinal (GI) bleeding.

## Case presentation

**Figure 1:**
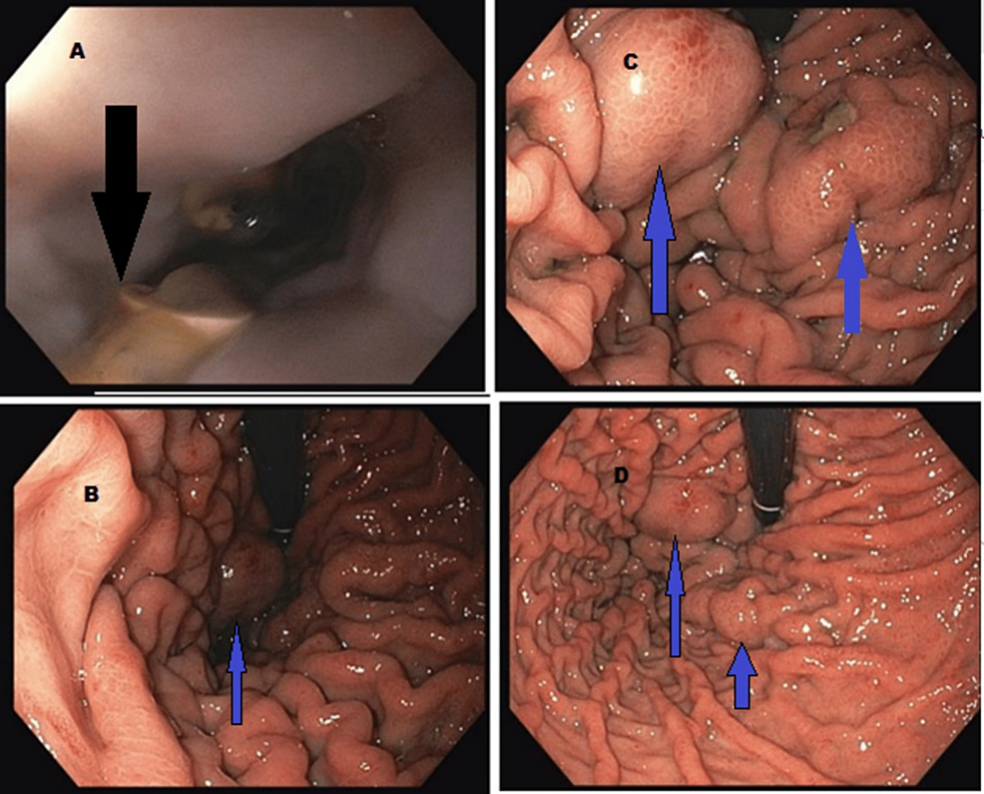
A - Indicates post-banding esophageal ulcers (black arrow). B, C, & D - Indicate gastric varices (blue arrows)

A 56-year-old male was admitted with recurrent upper GI bleeding (UGIB) with recent endoscopy (EGD) showing the presence of GV and EV. The patient had a past medical history of decompensated nonalcoholic steatohepatitis cirrhosis, diabetes, and hyperuricemia. The EV was treated by band ligation. However, the patient developed melena. The patient was hemodynamically stable with a hemoglobin and hematocrit (Hb/Hct) 11.3/33.3, international normalized ratio (INR) 1.5, platelet 126 (baseline around 120s), aspartate aminotransferase (AST)/alanine transaminase (ALT) 48/34, bilirubin 5.7, and albumin 3.1. He was started on an octreotide infusion, fluid replacement, intravenous pantoprazole, and ceftriaxone. The EGD showed multiple large post-banding esophageal ulcers (Figure 1A) as well as multiple medium and large GV (Figures 1B-1D). Endoscopic ultrasound (EUS) was used to inject the glue into the GV.

**Figure 2:**
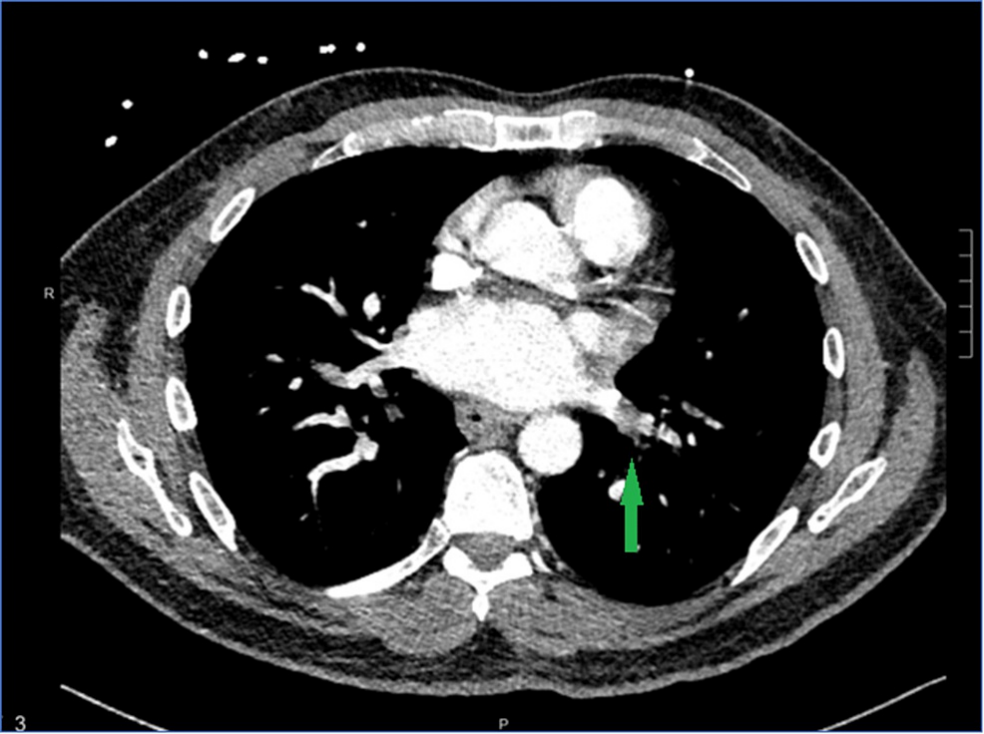
Sub-segmental PE (indicated by green arrow) PE: pulmonary embolism

The day after the procedure, the patient started to experience shortness of breath and was found to be hypoxic with oxygen saturation in the 80s with minimal physical activity. Given the recent injection of glue into the GV, a GPE was suspected as the potential cause of these findings. CT angiography of the chest showed two sub-segmental NGPEs (Figure 2). The presence of NGPE was confirmed by two different radiologists.

A duplex scan of the lower limb was negative for deep vein thrombosis (DVT). Given the presence of symptoms secondary to sub-segmental PE, the anticoagulation team was consulted about a recommendation regarding the potential use of anticoagulation (AC) versus surveillance due to the risk of bleeding. After multidisciplinary discussions, the decision was made to start AC with a low-dose heparin infusion, which was later switched to therapeutic enoxaparin for three months. The patient remained stable on AC without any bleeding with a stable Hb/Hct. The patient was discharged with instructions to follow up closely with outpatient gastroenterology and primary care physician.

## Discussion

Gastric varices are a known complication of DLC. There are two types of GV: isolated GV (IGV) and gastroesophageal varices (GOV). There are various treatment options for GV, including endoscopic or radiological approaches. The endoscopic approach may include band ligation, glue injection, EUS-guided coil with glue injection, cyanoacrylate injection, or a combination of these approaches [5]. Treatment of the GV can be associated with mild complications, such as fever, retrosternal or abdominal pain, or ulceration, or severe complications such as perforation and mediastinitis [5].

Glue pulmonary embolism is an infrequent complication following endoscopic glue injection for the treatment of GV. The risk factors for the development of GPE include the size of the varix, the speed of injection, and the volume of the injection [6]. However, in our patient, the PE following the management of GV was found to be NGPE, which was confirmed by two separate radiologists. CT angiography of the chest shows radiopaque material in the pulmonary vessels in the case of GPE rather than a filling defect, which is seen in the case of NGPE [7].

Patients with DLC have hypercoagulable changes such as hyperactive platelets, von Willebrand factor (VWF)/ADAMTS13 imbalance, and a hypofibrinolytic state [2]. Some studies have shown that lower albumin alone can be considered a relative risk factor for the development of venous thromboembolism (VTE). Prolonged hospital stay and immobility have also been established as common risk factors for VTE [2]. A meta-analysis of 11 studies involving 695,012 patients with hepatic cirrhosis showed an increased risk of thrombosis-related events when compared with 1,494,660 patients without hepatic cirrhosis (controls) (OR: 1.703; 95% CI: 1.333, 2.175; P < 0.0001) [8]. In a small randomized controlled trial on cirrhotic patients, a 12-month course of enoxaparin decreased the incidence of portal vein thrombosis, delayed decompensation, and increased survival rates [9]. However, to this moment, thromboprophylaxis in cirrhotic patients has not been adopted in guidelines and is not widely used in cirrhotic patients due to the perceived increased risk of bleeding [2]. The benefits of using the AC may be more than the risk of recurrent upper GI bleeding. Using AC after a recent UGIB is challenging. The literature suggests that reinitiating AC at discharge after an interruption for UGIB during hospital stay did not significantly increase the risk of recurrent UGIB within 90 days. It was also found to have a smaller number of associated thrombotic events [10]. This study, however, was not done in cirrhotic patients.

Recent guidelines recommend low molecular weight heparin as the treatment of choice for DVT or PE in cirrhotic patients with Child-Pugh B or C. Unfractionated heparin is the treatment of choice in patients with renal failure. In patients with Child-Pugh A, direct oral AC can be considered. Warfarin usage is limited due to alteration in the INR level in cirrhotic patients [2]. The development of NGPE after injection of glue for variceal treatment highlights that NGPE should still be considered in patients who develop respiratory complications after glue injection. This is important to identify, as AC is not indicated in GPE, but is required in the management of NGPE.

## Conclusions

Pulmonary glue embolism can occur as a complication from the treatment of gastric varices. However, NGPE should still be considered in patients who develop respiratory symptoms after the injection of glue in gastric varices. An NGPE needs to be treated with AC in contrast with observation for GPE.
